# The logic of basic education provision and public goods preferences in Chinese fiscal federalism

**DOI:** 10.1371/journal.pone.0225299

**Published:** 2019-12-02

**Authors:** Alfred M. Wu

**Affiliations:** Lee Kuan Yew School of Public Policy, National University of Singapore, Singapore, Singapore; Southeast University, CHINA

## Abstract

Without election or re-election motivations, what factors have impacted public goods preferences in an authoritarian country such as China? More specifically, what makes political elites be devoted to or not be devoted to local public goods provision? This study, using basic education provision as an example, intends to gauge the impact of leadership selection on public goods provision in China. It is found that career trajectories of politicians have a bearing on basic education provision. The findings suggest that even under a top-down appointment system, homegrown politicians are more willing to cater to local preferences, especially on basic education provision, which suggests an extension of Riker’s theory, applied in a non-democratic regime. Numerous studies have examined the impacts of decentralization on a variety of aspects of public governance in different contexts. Nevertheless, the unique contribution of this study is its policy implication that political centralization may not be an effective solution for local public governance even in an authoritarian context.

## Introduction

There is a small but growing debate about the relationship between leadership selection and public goods provision [1–3]. When examining the effects of fiscal federalism, Riker argued that local leaders appointed by high authorities are detrimental to both public goods provision and local accountability. Elected political elites are instead helpful in improving local public goods [4].

Though Riker’s theory is influential [5], the empirical test of Riker’s theory is still limited and the findings are mixed. Some confirm the theory: political decentralization wherein people elect their governors generates improved government quality and public services (for example, see the reference [6]). Nonetheless, Enikolopov and Zhuravskaya, drawing from a dataset of 75 developing countries for 25 years, find that top-down appointment or bottom-up election does not affect public goods provision [7]. Some even suggest a positive relationship between political centralization and public goods provision. Gennaioli and Rainer document a significant and positive relationship between the centralization of local chiefs and public goods provision such as education, health, and infrastructure in Africa [8] (see the reference [3]). It means that through enhanced accountability, the centralization of local chiefs may have a positive impact on public goods provision.

The theoretical linkage between political centralization and public goods provision could be debatable. It is worth investigating in a non-democratic regime as Riker’s theory was developed in a democratic setting. Furthermore, extending Riker’s theory–local leaders appointed by the central government (centralists) would not attend to some public goods provision which may not be visible to the central leaders, who could determine local leaders’ careers. However, localists who have developed their careers locally, although appointed by the central government could be incentivized to provide better public goods provision, such as education and health care, as they could benefit from the improvement of public goods personally. Some existing literature has documented the incentive structure of centralists and localists in the Chinese context. For example, centralists tend to promote central policies wholeheartedly while localists might twist central policies to maximize local interests [9]. Anecdotal evidence suggests a relationship among leadership selection, political centralization, and public goods provision. Nevertheless, empirical evidence based on a rigorous statistical analysis would shed fresh light on the literature and help generate some policy implications beyond the Chinese context. Though, internationally, evidence on the relationship between leadership selection and public goods provision is mixed, it is important to investigate whether Riker’s theory could be applied to an authoritarian context such as China. All regional leaders are appointed by higher authorities in China. For example, both provincial party secretary and provincial executive are selected and appointed by the central government. Previous research suggests that politicians’ career trajectories matter in affecting government behavior [9, 10]. We assume that leaders who develop their careers at the central ministries have different career paths from those who advance political careers locally, thus resulting in varied governing approaches.

The aim of this study is to develop an extension of Riker’s theory in an authoritarian context, which has rarely been done before. China provides an ideal venue for extending Riker’s theory despite his conjecture appearing to be more relevant in Western democracies. We argue that the rationale behind Riker’s theory, nonetheless, is applicable to an authoritarian context. Though China is a *de jure* unitary country, some characteristics of both central-local relations and regional leadership selection are parallel to the Western ones [11]. A *de facto* federalism has been adopted in the country [12, 13]. With a huge geographical size but one administrative system, the Chinese experience can be viewed as a natural experiment for testing the incentives affecting local public goods provision.

Our empirical evidence shows that regional leaders advancing their careers largely at the central government (centralists) tend to pay less attention to local public goods provision such as education. Homegrown politicians (localists) are more interested in promoting public goods provision, which benefits both the general public and themselves.

This study makes several contributions. First, it extends Riker’s theory in an authoritarian context. Though local capture may work to hinder homegrown politicians from improving local public goods, the empirical finding we obtained suggests that although appointed by the central government, localists are beneficial to local basic public goods such as education, as they are incentivized to improve its provision. Second, by utilizing sub-provincial data on fiscal decentralization to which scholars pay increasing attention [14–17], this study will advance the understanding of regional public governance in a rapidly changing society, where local governments attempt to adapt to specific circumstances while remaining loyal to the central government. Third, it casts doubt on the benefits of political centralization in China, which have been reiterated by some literature explaining the economic performance of certain transitional countries [18]. Evidence in our paper suggests that political centralization with many political elites sent by the central government to local governments, may not be an effective solution to improve local public goods provision and local governance.

The rest of the paper is presented as follows. Section 2 reviews the leadership selection, local public goods provision, and basic education in China. The main hypothesis is presented in this section. Section 3 introduces the empirical strategy for this study while Section 4 discusses the empirical finding on leadership selection, basic education provision, and public governance in China’s local state. Section 5 concludes with policy implications.

## Local leadership, *nomenklatura*, and local public goods in China

The “*nomenklatura*” is central to leadership selection in China. Based on the Leninist principle of organizing state and society, the *nomenklatura* widely applied in Communist countries refers to “a list of positions, arranged in order of seniority, including a description of the duties of each office” ([19] p.494). Parallel to its counterpart, the Chinese “*nomenklatura*” is a “leadership selection system that gives territorial party committees at each administrative level, monopoly power to select officials for posts within their jurisdiction…Each party committee from the Central Committee [of the Chinese Communist Party (CCP)] on down has a list of positions over which it has final selection authority” ([20] p.34). There are 2, 500 officials with the rank of governor and minister, and 39,000 officials with the rank of bureau controlled by the Central Committee’s *nomenklatur*a list ([20] p.34).

Vertical and horizontal interactions among leaders result in a nuanced picture of central-local political relations in China. According to the “*nomenklatura*” system, the central government selects provincial leaders on the one hand; the top Party leaders in the country, on the other hand, also need the support of regional leaders through the voting mechanism of the Central Committee of the CCP. Shirk thus calls it “reciprocal accountability” within the CCP [21]. Based on the institutional arrangement, the Chinese personnel system appears to be more complex than expected.

The central appointment does not mean identical political incentives across regions. Compliance with central directives is also not guaranteed at the local level. Local noncompliance or even defiance takes place in reality. The examples of local variations in responding to central policies are not rare in the history of the People’s Republic of China (PRC). For example, regional leaders’ reactions varied greatly during post-Mao decollectivization. Some local leaders were zealous to promote decollectivization in rural areas, whereas some ostensibly resisted the demands of the central government [22]. Even though the central government has a final say over the selection of provincial leadership, some local leaders are able to bargain for a preferential arrangement in many cases. For example, Ye Xuanping, the son of Marshal Ye Jianying, was renowned for his defense of local interests. When the central government intended to displace him in 1991, Mr. Ye negotiated with the central authorities and was finally able to turn his client into his successor. It is generally argued that leaders who have developed their careers locally tend to protect local interests in the first place [23].

Previous literature suggests that those formerly working in central ministries or holding a position in the central government concurrently—centralists—are more likely to align local interests with central ones. Furthermore, central policies are better promoted by local governments controlled by centralists (see [9, 24, and 25]). Homegrown politicians in contrast prefer programs which are instrumental to local interests and benefit ordinary people. Persson and Zhuravskaya report that Chinese local party secretaries who had developed their careers within the jurisdiction where they governed tended to promote better public goods provision than those having their careers elsewhere [2]. In addition, homegrown regional leaders are expected to be less predatory toward the private sector in China [2].

We thus hypothesize that centralists have a lower probability of boosting local public goods provision, such as basic education, while localists have a higher probability of promoting local public goods.

As economic development is an overriding concern of the Chinese central government, centralists prefer to spend more on infrastructure, and less on education and health provision due to political career concerns [10]. Similar to the practice in the Western democracies [26], economic growth is a visible, signaling device to improve governors’ chances of being promoted in an authoritarian context. In addition, short-term horizons associated with short tenure length (roughly 3.53 years between 1978 and 2004, see the reference [27]) render centralists less likely to invest resources in long-term projects such as education improvement. However, those officials deriving their prestige and power from their constituencies tend to cater to local preferences because they spend nearly all their time in the localities. Furthermore, homegrown politicians have fewer opportunities to be promoted to higher authorities [23]; thus, enhancing public services may add to the benefits of these “marginalized” senior officials. By all accounts, homegrown politicians may have incentives to promote local public goods.

Basic education has been selected as an example of local public goods in China. Two reasons stand out. First, basic education plays an essential role in improving the local economy and public governance in China. In the pre-reform period, education was depicted as an instrument to promote the Communist Party ideology and consolidate the CCP’s power. Schools and the education bureaucracy were empowered by the Party to shape people’s mindsets through imposing carefully-designed curricula on students. After the economic reform in 1978, the ideology concern has been no more at the forefront of social life and education [28]. Nevertheless, the importance of education has not reduced but strengthened. As a middle-income country, the Chinese government has well recognized the essential role played by education in promoting human resources and economic growth. Equally important is social cohesion brought by improved education in both urban and rural areas [29]. As early as 1986, the nine-year compulsory education law of the PRC was passed. The Chinese central government has introduced numerous measures to promote universal compulsory education across the board [29]. In recent years, in light of the importance of basic education in China’s local state, the Chinese government, especially the central government, has poured substantial financial resources into the education sector. A variety of programs such as the free Rural Compulsory Education program have brought enormous changes to Chinese society and economy [30, 31].

Second, education constitutes the largest category of public expenditure in China’s local state. According to the World Bank, basic education is also the largest part of public service units in China with private providers playing a supplementary role in the area [32]. In recent years for example between 2007 and 2011, education spending roughly ranged 16 percent-18 percent of local government budgetary expenditure. It ranked the highest among all spending items. The payroll of the basic education sector is also much larger than that of regular civil servants in China’s local state. Thus, basic education provision is of great significance to local public finance and public goods provision.

Development theory proposes the central role of basic education in developing countries. The Chinese practitioners have introduced “Education first” (prioritizing basic education in local decision-making, *jiaoyu xianxing*) in a bid to reduce poverty, improve human capital, and even build a harmonious society. Variations among regions, nonetheless, are by no means low. For example, if using the indictor of the teacher population, the number of teachers in basic education as a share of the total population, one will find that some provinces such as Xinjiang had 11.23 teachers per 1,000 inhabitants while Sichuan Province, also in the Western part of China, only recorded 4.74 teachers per 1,000 inhabitants between 1995 and 2007. The number in Zhejiang Province was 6.68 while the neighboring province, Fujian, had 8.75 teachers per 1,000 inhabitants in the same period. As basic education is a transaction-intensive industry, the number of teachers in a given region is vital to the performance of the education system in the area.

This begs the questions: why do some regions perform well in providing local public goods and others do not? To what extent does leadership selection—centralists or localists—affect public goods preferences? Answering these questions may be instrumental for us to have a nuanced understanding of local public goods provision in an authoritarian context.

## Econometric analysis

We used random-effects (RE), fixed-effects (FE), and two-stage least squares (2SLS) estimations for this study. Several robustness checks were implemented. A panel dataset of 29 provinces in China between 1995 and 2007 was used (Tibet was excluded and Chongqing was merged into Sichuan Province). Table 1 shows the details of variable definitions, descriptive statistics and data sources. Some key data about public finance was retrieved from the Compendium of Fiscal Statistics for All Prefectures, Cities, and Counties (*Quanguo dishixian caizheng tongji ziliao*) while other main data came from the China Compendium of Statistics 1949–2008 (After 2008, the Ministry of Finance of the PRC no longer published the compendium.

**Table 1:** Variable definition, descriptive statistics and data source, 1995–2007.

Variable	Definition (Mean; Std. dev.)	Data source
Public goods provision (Teacher population share)	Teachers in primary and regular secondary schools per 1,000 population (8.117; 1.306)	China data online
Bureaucratic Integration	See pages 16–17. (1.994; 0.648)	Wang and Ren (2009)
Expenditure decentralization within province	Expenditure at municipal, county and township levels as % of provincial expenditure in aggregate (70.852; 10.339)	Compendium of Fiscal Statistics for All Prefectures, Cities, and Counties (*Quanguo dishixian caizheng tongji ziliao*)
Revenue decentralization within province	Revenue at municipal, county and township levels as % of provincial expenditure in aggregate (77.801; 12.634)	Compendium of Fiscal Statistics for All Prefectures, Cities, and Counties (*Quanguo dishixian caizheng tongji ziliao*)
Economic development	Real GDP per capita (2476.786; 1750.787)	China compendium of statistics 1949–2008
FDI share	FDI as % of GDP (3.611;3.600)	China compendium of statistics 1949–2008
Population density	*De facto* resident per sq. km (367.381; 417.893)	China compendium of statistics 1949–2008
Student population share	Students in primary and regular secondary schools per 1, 000 population (151.416; 36.378)	China data online
SOE employment share	The numbers of SOE (state-owned enterprise) employees as % of local population (7.918; 4.439)	China compendium of statistics 1949–2008
Share of Secondary Sector in GDP	The volume of the secondary sector in GDP (44.937; 7.560)	China data online
Urban Unemployment rate	Registered unemployment rate in urban areas (3.506; 1.017)	China Statistical Yearbook

### Dependent variable

This paper employs teacher population share as a proxy of basic education provision. More specifically, the dependent variable is measured by the number of teachers in primary and regular secondary schools per 1,000 inhabitants in a given region.

The measurement of local public goods provision in the Chinese context has been of great complexity. Public spending on education, health care, or road infrastructure was ever used in the Chinese context [33, 34]. Researchers use budgetary data with caution when studying public goods and services in China. There are some problems pertinent to utilizing this data. Public expenditures on education and health care and those on culture and science are put together in budget tables in China. In addition, the category on public spending on education and health care has altered substantially over the past three decades, thereby, making these data less comparable. Perhaps more importantly, parents’ contributions, for example, play a significant role in financing education in China—it is almost the same in terms of private expenditure on basic education in many developing countries [35]. Public allocation to education therefore can hardly represent the actual level of public services in the education sector.

We acknowledge the complexity and difficulty of finding a suitable proxy for basic education in an authoritarian and developing context. Nevertheless, teacher population is an acceptable indicator for measuring basic education, involving face-to-face delivery of public goods, in China. As a transaction-intensive sector, the quality of education largely depends on the teacher population. Thus, it is not surprising to see that the Chinese central government continuously reiterates the importance of teacher population in improving public goods provision at the local level [36]. In addition, provincial executives have a great leverage over education policy especially at the primary and secondary levels. According to the Ministry of Education, though teacher-student ratio is prescribed by the national government, provincial governments have the responsibility to come up with their own policies in accordance with local circumstances [36]. The linkage between provincial leadership and teacher population therefore would be visible.

### Independent variables

#### Bureaucratic integration

This variable indicates the political incentives of regional leaders including provincial party secretaries (*shengwei shuji*) and executives (*shengzhang*). We suppose that regional leaders, who have accumulated their political assets in the central government before they become local leaders, have a higher possibility of promoting the interests of the central government. Those who built their careers in local jurisdictions have been more interested in enhancing local public services. Bureaucratic integration ([9] pp. 210–211) provides a proxy for measuring the preferences of regional leaders based on the data on career paths of both provincial party leaders and executives [10, 24, and 25].

Bureaucratic integration is assigned the number 4 when a provincial leader held a central government post concurrently (such as Politburo member). It has a value of 3 when provincial leaders had served in central ministries for at least 3 years before their appointments as provincial leaders. A value of 2 means that these leaders had worked in other regions prior to their current appointments while 1 denotes that local leaders only served in a given region for a long period. It is hypothesized that a higher value of bureaucratic integration is associated with a smaller teacher population share.

**Fig 1:**
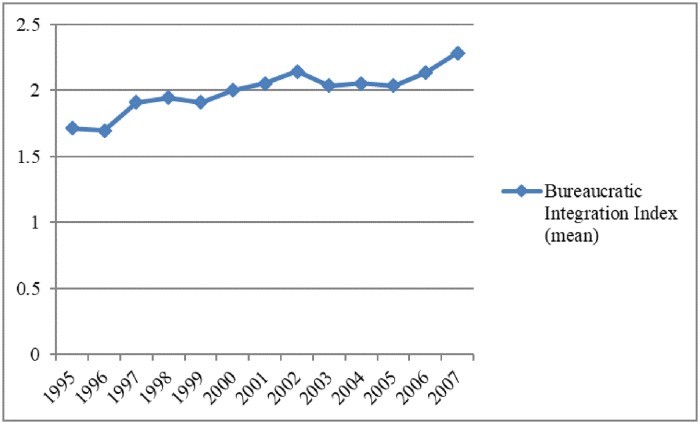
Bureaucratic integration (sample average), 1995–2007. Sources: the author with reference to [10].

Fig 1 shows an upward trend of bureaucratic integration with regard to local provincial leaders between 1995 and 2007. The year 2000 was a turning point when the value of bureaucratic integration reached 2. The years 2002 and 2007 saw the highest levels of bureaucratic integration. The 16^th^ and 17^th^ National Congresses of the Chinese Communist Party (CCP) took place in 2002 and 2007 respectively when personnel decisions were announced. It appears that new central leaders might intend to consolidate their power by improving the share of centralists in local governments.

#### Fiscal decentralization

The impact of fiscal decentralization is debatable. Addressing the informational problem between the principal and the agent, decentralization is supposed to have a positive impact on basic education provision [37]. Some evidence nonetheless, suggests a different story. A negative consequence of fiscal decentralization on government expenditure on education using the Chinese case has been observed [38–40].

A note is needed for the measurement of fiscal decentralization in the Chinese context. Probably due to the influence from cross-national research on decentralization, fiscal decentralization in China has usually been measured by the share of regional expenditure or revenue to the national aggregate [41, 42]. As the value of the denominator is constant in a given year within the country, the local share of total expenditure or revenue actually reflects the absolute value of expenditure or revenue in a given province (cross-national data suffers less). Oates cautions again this measurement despite applying it [43]. Though the above measures are not seriously flawed, an alternative way can be employed. We use the ratio of sub-provincial expenditure/revenue to provincial expenditure/revenue as the measure of expenditure and revenue decentralization [44]. Sub-provincial expenditure/revenue refers to public expenditure or revenue of prefectural, county, and township governments. Provincial expenditure/revenue sums up all finance activities at four tiers of government in China’s local state. It should be noted that there are five tiers of government in China: central, provincial, municipal, county, and township. Given the bulk of public services such as education are provided by sub-provincial governments, this measurement of fiscal decentralization across regions is much more revealing than the traditional one.

#### Socioeconomic characteristics

We introduce some variables to enhance model robustness. First, economic development is incorporated. Economic development is widely expected to have an impact on public goods provision. We hypothesize that economic development is positively associated with public goods provision because wealthier regions have greater capacity to improve local education. Economic development is measured by real provincial GDP per capita. Personal income is an alternative consideration (for the limitations of income and wage data in China, see the reference [45]); nevertheless, the GDP indicator is commonly used in relevant studies [41, 46] in part due to data quality and data availability with regard to personal income in China.

Second, economic openness is measured by the ratio of foreign direct investment (FDI) to local GDP. In many developing countries, FDI is central to both wealth accumulation and government performance. The amount of FDI inflow is used to measure local leaders’ performances by higher authorities in China; thereby local governments have an incentive to improve soft environments for attracting FDI. In the context of Vietnam, Malesky finds that public governance in local Vietnam is positively associated with FDI inflow [47]. We propose that FDI inflow improves public goods provision. A caveat is in order. Provinces with greater FDI inflow may coincide with more rural-to-urban migrant workers who would like to take up job opportunities in foreign direct investment factories. Given the Chinese local governments widely practice hukou-based public goods provision which is not in favor of rural immigrants, FDI inflow may be negatively associated with basic education provision as local governments are likely to ignore the demands of migrant workers. In a nutshell, the Chinese context-specific characteristics may complicate the association between FDI and public goods provision.

Third, population density is measured as the number of *de facto* inhabitants per square kilometer of land area. Some empirical evidence suggests that scale effects exist in the provision of public goods [48, 49] whereas others argue that there is a more complicated pattern [42]. We hypothesize that larger population density is associated with lower teacher population share, other things being equal.

Fourth, student population share is measured by the number of students in primary and regular secondary schools as a share of the local population. In basic education, students are the most important stakeholder. There is an official regulation of the teacher-to-student ratio in China. A class size of 45 to 50 students in secondary education should correspond to three teachers. Meanwhile, a class size of 40–45 students in primary education must have 1.5 teachers [36]. We expect that student population share is positively associated with teacher population share.

Fifth, SOE (state owned enterprise) employment as a share of population is included. Parallel to other Communist countries, SOE employment has been the dominant form of employment among the urban population in China. SOEs provide comprehensive benefits—including traditional public goods which ought to be provided by the government—to their employees; a higher teacher population is expected in areas with a state-dominated economy. Some anecdotal evidence also suggests that teacher population is extraordinarily high in areas hosting large state-owned enterprises [50]. We expect that larger SOE employment is associated with higher teacher population.

Sixth, two indictors—share of the secondary sector (manufacturing) in local GDP and urban unemployment rate—are introduced as control variables. It should be noted that the latter is the officially registered unemployment rate in urban areas; we suspect that the available data has a systematic downward bias.

### Model specifications

We adopt panel data models in our study. Through “blending inter-individual differences and intra-individual dynamics,” panel data can help untangle the complexity of social phenomena [51]. The fixed-effects models always generate consistent results; however, they may not be the most efficient models. The random effects models instead can generate consistent and efficient results; therefore, as long as a random effects model is statistically justified, it is preferred over a fixed effects model [52]. The Hausman specification test is commonly used to compare fixed effects and random effects. In this study, the Hausman test statistics suggest that fixed effects models are preferred though they are less efficient.

In a bid to investigate local public goods provision across provinces in China, the basic panel data model we apply is as follows:

$$Y_{jt}=\alpha_{0}+{\beta X}_{jt}+{\gamma C}_{jt}+\alpha_{j}+\gamma_{t}+\varepsilon_{jt}$$

where *j* indicates provinces, *t* denotes time, *Y* refers to local public goods as measured by the share of teacher population in the total population, X denotes two other important variables—bureaucratic integration and fiscal decentralization while C refers to a battery of socioeconomic variables, *α*_*j*_ and *γ*_*t*_ refer to the province-fixed effects and the year-fixed effects, respectively while *ε*_*jt*_ is the random error.

The models used in this study raise issues about reverse causation between teacher population share and economic development. Teacher population can contribute to GDP in various ways. Since we are interested in the one-way relationship between the dependent variable and independent variables, we need to deal with the endogeneity problem. A two-stage least squares (2SLS) estimation is therefore employed. We enter lags two to three of the regressors (GDP) as instruments to handle the endogeneity threat (Column 3–6 of Table 2). The instruments pass the weak instrument test [53]. Furthermore, the Sargan statistics in the overidentification test also suggest that instruments which we adopted are valid [54]. All tests suggest that the null hypothesis of no correlation between the instruments and the error term in the regressions cannot be rejected, indicating that the instruments could be considered to be exogenous.

**Table 2:** Determinants of basic education provision in China, 1995–2007.

	FE (1)	FE (2)	2SLS (3)	2SLS (4)	2SLS (5)	2SLS (6)
Bureaucratic Integration Index	-0.140* (0.080)	-0.142* (0.082)	-0.147** (0.073)	-0.148** (0.072)	-0.147** (0.073)	-0.149** (0.073)
Expenditure decentralization within province	-0.003 (0.009)	-0.004 (0.009)	-0.003 (0.009)	-0.003 (0.010)	-0.003 (0.009)	-0.003 (0.010)
Revenue decentralization within province	0.018 (0.026)	0.018 (0.026)	0.017 (0.021)	0.018 (0.021)	0.017 (0.021)	0.018 (0.021)
Economic development	2.195** (1.050)	2.187** (1.021)	1.598 (1.193)	1.528 (1.162)	1.564 (1.174)	1.490 (1.153)
FDI as a share of GDP	-0.047* (0.028)	-0.046* (0.027)	-0.045** (0.019)	-0.044** (0.018)	-0.044** (0.019)	-0.044** (0.019)
Population density	4.620 (3.874)	4.658 (3.710)	5.193* (2.952)	5.227* (2.797)	5.226* (3.018)	5.260* (2.850)
Student as a share of population	0.002 (0.002)	0.002 (0.002)	0.002 (0.001)	0.002 (0.001)	0.002 (0.001)	0.002 (0.001)
SOE employment as a share of population	0.279*** (0.052)	0.282*** (0.048)	0.276*** (0.036)	0.278*** (0.032)	0.276*** (0.036)	0.278*** (0.032)
Share of secondary sector in GDP		-0.002 (0.015)		-0.002 (0.010)		-0.002 (0.010)
Urban unemployment rate		0.001 (0.081)		-0.005 (0.067)		-0.005 (0.067)
Constant	-37.688 (23.972)	-37.678* (21.948)				
R-squared	0.4214	0.4212	0.4206	0.4203	0.4205	0.4202
N	336	334	336	334	336	334
Cragg-Donald F Statistic			229.969	221.959	156.177	150.457
Hansen J statistic (p-value)			0.7632	0.7547	0.9510	0.9453

*** p<0.01,

** p<0.05,

* p<0.1;

Standard errors in parentheses; 3,

Economic development and population density are in logarithms;

Models 1 and 2 report fixed effects results; Models 3 and 4 report 2SLS results with GDP lagged by 2 years as an instrument while models 5 and 6 report 2SLS results with GDP lagged by 3 years as an instrument.

### Empirical results

Table 2 presents the result of our panel data analyses. The VIF tests indicate there is no multicollinearity problem in all of the models we employed. Model 1 includes major variables including bureaucratic integration, expenditure/revenue decentralization, economic development, FDI share, SOE employment, population density, and student population share. The R-squared of 0.42 suggests that the model can explain roughly 42 percent of the variation of the dependent variable. Model 2 adds two more socioeconomic variables, namely economic structure (secondary sector output as a faction of GDP) and urban unemployment rate. With fewer observations, Model 2 can also explain 42 percent of the variation of the teacher population share. Economic development (GDP) might be endogenous. Teacher population, as the number of public sector employees paid out of the government coffer, could contribute to GDP growth. We further include Model 3 and Model 4 with real GDP lagged by 2 years and Model 5 and Model 6 with real GDP lagged by 3 years to account for endogeneity. All four models have a higher significance level with regard to bureaucratic integration.

Our key explanatory variable—bureaucratic integration—is significantly and negatively associated with the dependent variable though the 2SLS models suggest a higher significance level. In the fixed effects models, the significant level of bureaucratic integration is at the 10 percent level while it is at the 5 percent level in the 2SLS models. We also attempt to lag all independent variables by 2–3 periods. Bureaucratic integration is significantly and negatively correlated with teacher population share. In general, the coefficient estimates suggest that when bureaucratic integration shifts by 1 unit, teacher population share moves in the opposition direction by 0.140–0.149 units. Being one of the most populous countries in the world, the Chinese provincial population has a mean of 43.14 million in our study. As teacher population share is measured by the number of teachers per 1,000 inhabitants, the shift in the teacher headcount associated with changes of provincial leaders is substantial.

Fiscal decentralization neither on the expenditure nor revenue side has any explanatory power for public goods provision. The new measurement of fiscal decentralization can yield more meaningful empirical results because decentralization within a province reflects the dynamics of revenue sources and responsibilities of local public goods provision in China. Despite not being significant, the direction of the relationship between public goods provision and fiscal decentralization is reasonable. Revenue decentralization is positively associated with teacher population share, whereas expenditure decentralization is negatively related to the dependent variable.

Both the FDI share and the SOE employment share, exert influence over the dependent variable to some extent. The direction of the impact of FDI share, points to the story about hukou-based discrimination against migrant workers. Therefore, economic openness would not play a positive role in improving basic education provision. It means that regions with greater FDI inflow may not spend more on basic education; instead the regions spend less on this area in order to prevent outsiders including the children of migrant workers from enjoying basic education. SOE employment has a strong explanatory power in all the models. It confirms the legacy of the planned economy in public goods provision in transitional China. Before the large-scale privatization of SOEs in the 1990s, state-dominated economies usually had good basic education provision as the benefits were considered a part of employees’ fringe benefits. Though the comprehensive benefits offered by SOEs were dismantled gradually after the mid-1990s, the legacy still continues in state-dominated economies. In addition, many SOE employees become school teachers as the local state tends to accommodate former state workers.

Economic development and population density have significant impact on the dependent variable in the fixed effects models and the 2SLS models respectively while economic structure, student population share, and unemployment rate are not statistically significant in all the models. Though there is a statutory teacher-to-student ratio applied in China, there is no significant relationship between student population and teacher population in all the models.

Our empirical analyses generated the following results: 1) Riker’s theory holds true in the case of China. Regions governed by centralists tend to have poorer local public goods provision as measured by teacher population. 2) Fiscal decentralization has not exerted influence over local public goods provision statistically. Nevertheless, the estimated direction of the relationship between fiscal decentralization and local public goods provision are probable and consistent with previous studies. 3) Some control variables such as FDI inflow and the share of SOE employment play significant roles in shaping basic education in China.

## Discussion

Our empirical evidence shows that localists have the propensity to serve the people through improving basic public services such as education provision. The paper thus suggests that Riker’s theory can travel to non-democracies like China. Homegrown politicians, although appointed by the central government, tend to be beneficial to education provision in local China. A large body of literature has paid attention to both the Chinese cadre management system and the *nomenklatura* previously. Several researchers have pointed out that the cadre management system in China emphasizes the importance of economic achievements and revenue extractions rather than public goods provision such as education [55]. Therefore, provincial leaders coming from the central government are more eager to promote the policy that is favored by the central leadership, who in turn have the final say over the future promotions of provincial leaders [10]. In contrast, local public goods provision like basic education is more likely to be promoted by those who need to spend most of their time with local people. Our empirical evidence suggests that homegrown politicians prefer investing in basic education, which seems not directly related to economic performance.

This paper reveals that appointed regional leaders in an authoritarian country are not as homogenous as expected though they are governed by a single top-down personnel control system. In many cases, scholars have misread the effects of the above political centralization in China. When comparing Russia with China, some contend that political centralization distinguishes China as a successful transition economy. In contrast, the weak federal government in Russia rendered local governments very predatory. In a widely cited paper, Blanchard and Shleifer argue that “the central government [in China] has been in a strong position to either reward or punish local administrations, reducing both the risk of local capture and the scope of competition for rents” ([18] p.172). Nevertheless, “federalism [in Russia] has failed precisely because of political decentralization” ([18] p.177). Centered primarily on the Putin’s recent reform in Russia, counterarguments on political decentralization have emerged in the recent literature (for example, see the reference [56]).

The above argument may exaggerate the benefits of the central personnel control system on the one hand and neglect the heterogeneous governance structure in China on the other. To some extent, neither central nor local authorities are so benevolent towards their constituents in China [25, 57]. But homegrown regional leaders are more willing to promote basic public services such as education according to our empirical evidence.

## Conclusion

This study adds to the literature on the political logic of local public goods in an authoritarian context. The evidence suggests that bureaucratic integration is significantly and negatively associated with local public goods provision in China. The more integrated local politicians are with the central government, the lesser the possibility that they will promote basic education in China’s local state. Though our study deviates a little from the standard assumption about the incentives of regional politicians (elected or appointed) and public governance proposed by Riker, the Chinese case actually improves the explanatory power of Riker’s theory to some extent. It implies that even under a top-down appointment system, homegrown politicians may be more willing to cater to local preferences, especially on public goods provision.

This study therefore casts doubt on the benefits of political centralization as manifested in a central personnel control system in China. Compared with Russia, China’s transition from the planned economy seems more prosperous with regard to economic development. Some studies in the literature nonetheless overstate the benefits of Chinese institutional settings, especially of those concerning central-local relations [18]. A close scrutiny of central-local relations suggests that the intergovernmental political and fiscal relations in China result in numerous dysfunctions in the country. Local predatory behaviors, collusion among governments, and the like are rampant in Chinese bureaucracy [58–60]. Our study proposes additional evidence for the purpose of an up-to-date evaluation in this area. Policy implications drawn from this study for China and other developing countries are to improve the political incentives of local politicians in promoting basic public goods provision by aligning their careers with their performance in public governance. It is important to note that a follow-up, qualitative study may be needed to investigate the story in which officials with deeper and stronger local roots care more about basic education while officials who are more central policy-oriented tend to cater to visible economic achievements such as GDP growth.

Our second remark concerns the need to introduce the demand-driven approach in local public goods provision. The voluminous literature points out that some basic public goods provision is highly decentralized, especially for the case of basic education in China [29]. Decentralized basic education has nonetheless led to many negative consequences such as substantial regional inequalities in basic education. The World Bank even points out that the key issue in this area is to hold local governments accountable toward the central government [32]. Bottom-up components are ignored, however. A demand-driven approach in basic education provision, making service providers accountable to consumers, has received little attention in China. Centralization more often than not involves inefficiency and waste as central decision-making and local demands are often not linked together. Meanwhile, decentralization often leads to local capture wherein local elites benefit more from local public goods provision than ordinary people. Thus, empowering consumers is of more significance to local public good provision and local governance. Eckardt aptly points out that translating consumer demand into public expenditures is essential for improved public services in a developing context [61]. Evidence elsewhere suggests that the demand-driven approach can be combined with decentralized public goods provision to maximize the benefits to consumers [62]. In practice, nevertheless, consumers such as parents and students in China have rarely been provided enough power in the decision-making and the service delivery of local public goods. Improving consumers’ leverage in the basic education sector will not only change the landscape of local public goods provision but also affect local politicians’ public goods preferences. Politicians and bureaucrats may realize that economic achievements, revenue extractions, and soft infrastructure such as basic education will enhance their own and ordinary people’s interests.

## Supporting information

S1 File Data. (RAR)
